# CD248: A therapeutic target in cancer and fibrotic diseases

**DOI:** 10.18632/oncotarget.26590

**Published:** 2019-01-29

**Authors:** Beverly A. Teicher

**Affiliations:** ^1^ Molecular Pharmacology Branch, Developmental Therapeutics Program, DCTD, National Cancer Institute, Bethesda 20892, MD, USA

**Keywords:** CD248, endosialin, TEM1, sarcoma, fibrotic disease

## Abstract

CD248/endosialin/TEM1 is a type 1 transmembrane glycoprotein found on the plasma membrane of activated mesenchymal cells. CD248 functions during embryo development and is either not expressed or found at very low levels in adult tissues. CD248 is expressed at high levels by malignant sarcoma cells, by the pericyte component of tumor vasculature and by mesenchymal cells in some fibrotic diseases. CD248 is being targeted by several experimental therapeutics including antibodies, antibody drug conjugates, as an antigen for CART cells and in therapeutic vaccines. Although the function of CD248 has yet to be fully elucidated, this protein is a potential broad scope therapeutic target.

## INTRODUCTION

### CD248 protein, gene expression and regulation

CD248 is a transmembrane glycoprotein that is dynamically expressed on pericytes and fibroblasts during tissue development, tumor neovascularization and inflammation. In tissue remodeling, CD248 is associated with increased stromal cell proliferation and migration. CD248 may be useful as a molecular marker and therapeutic target for sarcoma and other diseases [1–14]. Endosialin/CD248/TEM1 was first identified in 1992 as the antigen of an antibody designated FB5 which was raised in mice inoculated with human fetal fibroblasts [10, 15, 16]. In tissues, FB5 reacted strongly with vascular cells in 67% of malignant tumor specimens and weakly with stromal fibroblasts in a subset of other specimens. This initial study provided evidence that CD248 was expressed during development, being a fetal antigen, that it was overexpressed in cancer tissues and that its expression varied between carcinomas and sarcomas. Subsequently, several reports of CD248 protein expression concurred that CD248 expression was limited to a few cell types in normal tissues and was mainly a developmental and pathologic feature. However, the CD248 transcript was found to be ubiquitously expressed in normal adult tissues and in somatic tissues during development, both in humans and mice [17]. Tissues with high CD248 transcript expressed the protein, while tissues with lower levels of the transcript were negative for the protein [18]. High CD248 was detected in fibroblasts and pericytes in human thymus, lymph nodes and spleen during lymphoid tissue development but was mostly absent in the adult except during secondary lymphoid organ remodeling during adaptive immune responses [19, 20]. In normal adults, endosialin protein expression appears to be limited to normal endometrial stroma and occasional fibroblasts [18, 21, 22].

### Binding partners

In addition to fibronectin and collagen, the metastasis-related protein Mac-2 BP/90K was reported to be a binding partner of CD248 [23]. Mac-2 BP/90K is weakly expressed in the stomach, small intestine, colon, kidney, and ovary but strong staining was observed on a panel of tumor tissues including carcinomas of the small intestine, renal cell carcinomas, and adenocarcinomas of the colon and uterus. In these samples Mac-2BP/90K was expressed exclusively by the malignant cells whereas CD248 was not detected in the cancer cells. Mac-2 BP/90K levels were elevated in the plasma of breast and lung cancer patients [24, 25]. A correlation existed between Mac-2BP/90K levels in circulation and the occurrence of distant metastasis suggesting that this ligand of CD248 could be prognostic biomarker.

**Figure 1:**
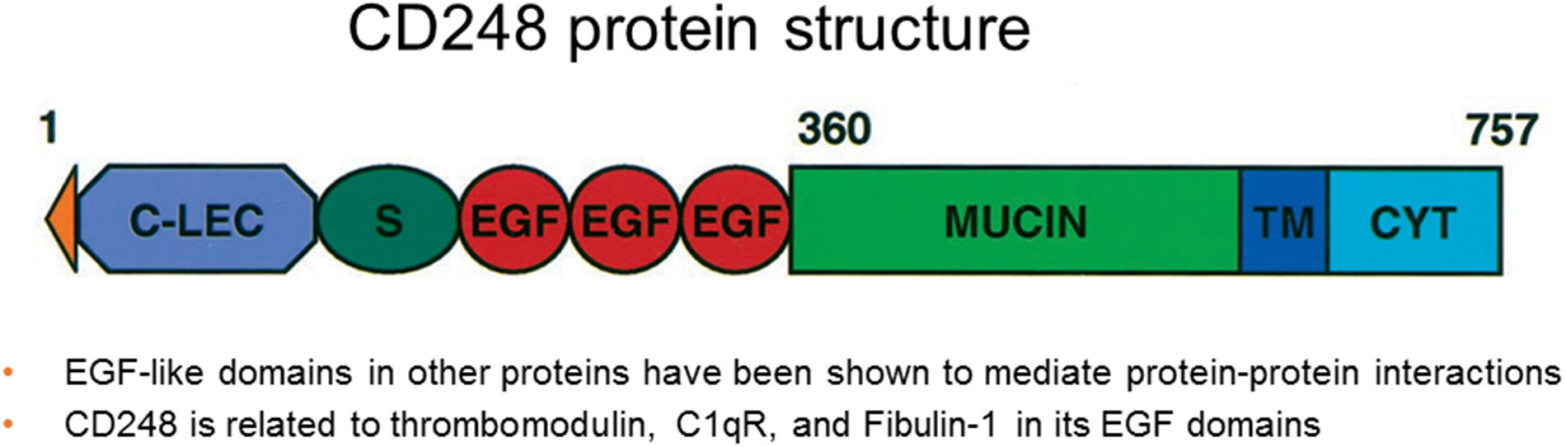
CD248 protein structure (adapted from Christian et al. JBC 2001; ref [56]

The CD248 protein sequence has EGF and thrombomodulin domains, suggesting a role in protein-protein interactions [26]. The molecular cloning of CD248 led to its further characterization as a type I cell surface glycoprotein of 757 amino acids with a predicted molecular mass of 80.9 kDa and one transmembrane domain [26, 27]. CD248 is classified as a C-type lectin-like protein comprised of a signal leader peptide, five globular extracellular domains (three of which are EGF repeats), a mucin-like region followed by the transmembrane region and a short cytoplasmic tail [28]. CD248 is most closely related to thrombomodulin/CD141 with 39% homology and 33% homology to the complement receptor C1qRp [6, 12]. Endosialin is localized on the q arm of human chromosome 11. The C-type lectin domain containing group 14 family members CLEC14A and CD93 are proteins expressed by endothelium and are involved in tumor angiogenesis [29]. CD248 is a member of this family and is expressed by some tumor-associated fibroblasts and pericytes. Multimerin-2 (MMRN2) is an endothelial selective extracellular matrix protein implicated in angiogenesis and tumor progression (Figure 1). CLEC14A, CD93 and CD248 can bind to MMRN2; however, thrombomodulin does not. Binding to MMRN2 is dependent on a long-loop region in the C-type lectin domain and is abrogated by mutation in that domain. While CLEC14A and CD93 bind to a non-glycosylated MMRN2 coiled-coil region, CD248 binds to a distinct MMRN2 region. CLEC14A and CD248 can bind MMRN2 simultaneously at the interface between endothelium and pericytes in human pancreatic cancer. A recombinant peptide of MMRN2 that blocks CLEC14A binding to endothelial is anti-angiogenic *in vitro* and in mice slows tumor growth [30].

Although CD248 protein structure has been characterized, CD248 function has not been fully characterized. Tumors generate areas of hypoxia due to inadequate delivery of oxygen. Hypoxia-inducible transcription factors, HIF-1 and HIF-2, are often upregulated leading to vascular remodeling [31, 32]. Ohradanova et al. demonstrated that CD248 gene transcription is regulated under hypoxic conditions via hypoxia-inducible factor 2 in placental fibroblasts and glioblastoma cells [33].

The murine ortholog of CD248 was cloned and found to be expressed during development and during implanted tumor growth in the adult mouse [34, 35]. In mouse embryos, CD248-lacZ co-localized with most vimentin-positive cells and a large portion of CD31- or desmin-positive cells. In the mouse, CD248 was expressed throughout embryonic and adult development in mesenchymal cells related to blood vessels [36]. Endosialin^-/-^ mice have no defect in pericyte recruitment, suggesting a role for endosialin in pericyte/endothelial cell cooperation during vascular patterning [3].Endosialin^-/-^ mice have higher than normal bone mass due to increased osteoblast-mediated bone formation [1]. Syngeneic tumors growth was slower in CD248^CyD/CyD^ mice, which lack the CD248 cytoplasmic domain. CD248^CyD/CyD^ fibroblasts have impaired PDGF-BB-induced migration, decreased matrix metalloproteinase (MMP)-9 secretion and tumor suppressors transgelin (SM22a), Hes and Hey1 transcript levels [6].

CD248 is involved in vascular angiogenic signaling and ECM components in tumors [37]. Cell surface expression may distinguish between mesenchymal stem cells (MSCs) from different sources, including bone marrow-derived MSCs and adipose-derived MSCs (AMSCs) grown in human platelet lysate. Although adipose-derived stromal cells survival in hypoxic grafts decreased over time, these cells provided multiple angiogenic growth factors, and therefore, improved fat graft retention due to better graft vascularization [38, 39]. The surface marker transcriptome of AMSCs, validated the expression of classical markers, and identified nine non-classical markers (CD36, CD163, CD271, CD200, CD273, CD274, CD146, CD248, and CD140B) that may potentially discriminate AMSCs [39]. Human AMSCs can be obtained in large quantities, are multipotent, and have trophic paracrine functions. AMSCs adhere to and can be cultured on surgical-grade porous titanium discs as a model for orthopedic implants and differentiated upon osteogenic induction. AMSCs grown in the porous titanium microenvironment compared with standard culture conditions displayed differences in temporal expression for genes involved in cell cycle progression (CCNB2, HIST2H4), extracellular matrix production (COL1A1, COL3A1), and mesenchymal lineage identity (ACTA2, CD248, CD44) [40].

## EXPRESSION DURING DEVELOPMENT AND PATHOLOGY

### Normal development and maturity

Stromal cell populations in lymphoid tissue express CD248 differentially on fibroblasts and pericytes in the thymus, lymph node and spleen. Expression is high during lymphoid tissue development and largely disappears in the adult. CD248 is re-expressed in a Salmonella-induced model of splenic enlargement; peak expression corresponding to the peak of splenic enlargement. Thus, CD248 expression helps define a subset of lymphoid tissue stromal cells which play a role in remodeling during tissue development, infection and repair [19]. Mesenchymal stem cells (MSCs) may be useful for treating degenerative or incurable diseases [109]. Microvessels from MSCs can contribute to recovery of damaged tissues in *in vivo* disease models. LC−MS/MS analysis of the microvessel proteome identified 730 proteins. Functional enrichment analysis showed that cellular processes represented by these proteins include cell proliferation, adhesion, migration, and morphogenesis. Integration of MSCs self-renewal and differentiation related genes and the proteome of MSC-conditioned media with the proteome revealed potential microvessel protein candidates that can be associated with the therapeutic effects: (1) surface receptors (PDGFRB, EGFR, and PLAUR); (2) signaling molecules (RRAS/NRAS, MAPK1, GNA13/GNG12, CDC42, and VAV2); (3) cell adhesion (FN1, EZR, IQGAP1, CD47, integrins, and LGALS1/LGALS3); and (4) MSC-associated antigens (CD9, CD63, CD81, CD109, CD151, CD248, and CD276 [41, 42]. CD248+ stromal vascular cells were analyzed using single cell transcriptional analysis to identify and cluster angiogenic gene-expressing cells, which were then correlated with surface marker expression. Stromal vascular cells isolated from human lipoaspirate were FACS sorted based on CD248. Cells were analyzed for angiogenic gene expression and ability to promote microvascular tubule formation *in vitro*. In mice, wounds treated with CD248+ cells healed faster, and at 7 days, had more re-epithelialization than wounds in other groups. CD31 immunohistochemistry showed higher vascularity in the CD248+ cells treated group at the time of healing and at 14 days, consistent with a pro-angiogenic effect of CD248+ cells [43, 44].

### Fibrotic diseases

CD248 is an activation marker of mesenchymal lineage cells including tumor-associated pericytes, stromal myofibroblasts, and activated vascular smooth muscle cells (VSMC). In rheumatoid arthritis and psoriatic arthritis, the synovium transforms from a thin pauci-cellular tissue into an invasive and joint destructive tissue that is characterized by hyperplasia, angiogenesis, immune and mesenchymal cell infiltration, and development of secondary lymphoid structures. Synovial tissue biopsy samples from healthy tissue, from psoriatic arthritis, and from rheumatoid arthritis indicated that synovium from psoriatic arthritis and rheumatoid arthritis and from mice after the induction of collagen antibody–induced arthritis stained strongly for CD248 in perivascular and fibroblast-like stromal cells. CD248 knockout (CD248KO/KO) and CD248 cytoplasmic domain lacking (CD248^CyD/CyD^) mice had less severe arthritis, with lower plasma levels of proinflammatory cytokines than controls. The joints of CD248^CyD/CyD^ mice had less synovial hyperplasia, reduced inflammatory cell accumulation, and less articular cartilage and bone damage. Tumor necrosis factor induced monocyte adhesion to CD248^CyD/CyD^ fibroblasts was impaired. CD248^CyD/CyD^ fibroblasts exhibited reduced HIF-1, PLGF, VEGF, and MMP-9 activity in response to TGF-*β*. CD248 may be involved in synovial hyperplasia and leukocyte accumulation in inflammatory arthritis [45]. Growth of T241 fibrosarcoma and Lewis lung carcinoma was slower in CD248^CyD/CyD^ mice. CD248^CyD/CyD^ fibroblasts secreted less MMP-9, had impaired PDGF-BB-induced migration and expressed higher transcripts of tumor suppressors, transgelin (SM22α), Hes and Hey1 [6]. The functional effect of CD248 genetic deletion on bone mass was investigated on the tibiae of 10-week-old wild-type or CD248 knockout mice and human and mouse primary osteoblasts. CD248 was expressed by human and mouse osteoblasts, but not osteoclasts. CD248 knockout mouse tibiae had higher bone mass and superior mechanical properties compared to control mice. Primary osteoblasts from CD248 knockout mice induced increased mineralization *in vitro* and produced increased bone over 7 days *in vivo*. CD248 knockout produced high bone mass due to increased osteoblast-mediated bone formation, suggesting targeting CD248 in rheumatoid arthritis [1]. Fibroblast-like synoviocytes were isolated from inflamed joints of mice expressing both the T cell receptor transgene KRN and the MHC class II molecule Ag7 (K/BxN mice). Fibroblast-like synoviocytes were identified by expression of fibronectin, prolyl 4-hydroxylase, CD90.2 and CD248 in >98% of the population. Fibroblast-like synoviocytes isolated from K/BxN mice had greater basal expression of inflammatory markers including IL-6, chemokine ligand 2 (CCL-2) and vascular cell adhesion molecule 1 (VCAM-1) compared to fibroblast-like synoviocytes isolated from non-inflamed tissue [46]. Synovial fibroblasts were cultured, and phenotypic changes followed upon exposure to interleukin-1*β*, TNF-*α*, and TGF-*β*1. In the lining layer in rheumatoid arthritis, synovial fibroblasts expressed higher podoplanin than normal synovium, and CD248 expression was restricted to sub-lining layer cells. TNF-*α* or IL-1*β* exposure increased podoplanin expression, while TGF-*β*1 exposure induced CD248 expression. In the SCID human-mouse model, rheumatoid synovial fibroblasts recapitulated the expression of podoplanin and CD248. Fibroblasts adjacent to cartilage expressed podoplanin, and attached to, invaded, and degraded cartilage. Podoplanin + CD248– synovial fibroblasts preceded the appearance of PDPN– CD248+ cells in contralateral implants. There were two distinct synovial fibroblast populations identified by expression of either podoplanin or CD248 which were located within different anatomical compartments of the inflamed synovial membrane [47]. Synovial tissue expression of stromal markers in early arthritis was analyzed using immunofluorescence to detect stromal markers CD55, CD248, fibroblast activation protein and podoplanin. Synovial fibroblast activated protein expression was higher in early rheumatoid arthritis patients. Podoplanin expression was highest in early inflammatory arthritis patients but did not differentiate diagnostic outcomes. Stromal cell markers CD55, CD248, FAP and podoplanin are expressed in the earliest stage of arthritis [48].

Kidney stromal fibroblasts produce fibrotic matrix. CD248 is expressed by key effector cells within the stroma of fibrotic kidneys including pericytes, myofibroblasts and stromal fibroblasts. To assess CD248 expression in kidney fibrosis and if it is associated with chronic kidney disease, CD248 expression was analyzed by immunohistochemistry in kidney biopsies. In normal kidney tissue, CD248 was expressed by pericytes, and occasional stromal fibroblasts. In human chronic kidney disease, expression was linked to known determinants of renal disease progression. CD248 was expressed by *α*-SMA-myofibroblasts and *α*-SMA-stromal cells but not CD45+ leukocytes. Thus, CD248 defines a subset of myofibroblasts linked to albuminuria and tubulointerstitial damage during tissue remodeling in chronic kidney disease [49–51]. Tissue fibrosis and microvascular rarefaction are hallmarks of progressive renal disease. In standard renal fibrosis models, CD248 knockout mice were protected from fibrosis and microvascular rarefaction due to a stabilizing effect of pericytes with less migration and differentiation of pericytes toward a myofibroblast phenotype. Thus, CD248 stromal cells have a role in renal fibrosis, furthermore, targeting CD248 was effective at inhibiting both microvascular rarefaction and renal fibrosis through modulation of pericyte and stromal cell function [50, 51].

Liver fibrosis is a reversible wound-healing response to injury reflecting the balance between liver repair and scar formation. Chronic liver damage leads to progressive substitution of liver parenchyma by scar tissue and results in liver cirrhosis. Stromal cells (hepatic stellate cells and endothelial cells) control the balance between liver fibrosis and regeneration. CD248 expressed in the liver by stellate cells and portal fibroblasts, was upregulated in liver fibrosis. Chronic chemical damage resulted in reduced fibrosis and enhanced hepatocyte proliferation in CD248 knockout mice. Acute-liver-damage-induced hepatocyte proliferation was increased in CD248 knockout mice. A candidate-based screen of known regulators of hepatocyte proliferation identified IGF-2 as a CD248-dependent hepatocyte mitogen. Thus, CD248 is a therapeutic target in fibrotic disease [52]. Normal and diseased human and murine liver tissue and isolated hepatic stellate cells were examined for CD248 protein and mRNA expression. Hepatic fibrosis was induced in CD248 knockout mice and control mice with carbon tetrachloride treatment. Little CD248 expression was seen in normal human and mouse liver but CD248 was increased in liver injury. Other fibroblast/stellate cell markers expressed in liver sections included desmin, vimentin and α-SMA. CD248 expression was restricted to isolated primary murine and human stellate cells. Collagen deposition and α-SMA expression, but not inflammation and neo-angiogenesis, was reduced in CD248 knockout mice compared with control mice after carbon tetrachloride treatment [53]. Inflammation is involved in the progression of tendinopathy. Stromal fibroblasts from patients with tendinopathy were analyzed for activation markers including podoplanin, CD106 (VCAM-1) and CD248. All three proteins were increased and persistent in diseased tendon tissues and in chronic inflammation and recurrent tendinopathy [54].

VSMC expressing CD248 are important in the pathogenesis of atherosclerosis. CD248 was upregulated during atherosclerosis in apolipoprotein E (ApoE)– null mice and human atherosclerotic samples. Atherosclerosis, assessed by descending aorta Oil Red O staining, was reduced in ApoE/CD248-deficient mice. CD248 was a regulator of VSMC phenotypic remodeling contributing to atherosclerosis [55]. The role of CD248 in idiopathic pulmonary fibrosis (IPF) was investigated using IPF lung samples and in cultured pulmonary fibroblasts and epithelial cells. CD248 silencing was evaluated on fibroblast proliferation and myofibroblast differentiation. CD248 was expressed in mesenchymal cells of normal lung structures such as pleura and adventitia but not in epithelium. Fibrotic areas had markedly stronger CD248 staining than normal lung. CD248 protein was higher in IPF-derived lung fibroblasts and CD248 silencing reduced the proliferation of lung fibroblasts but did not alter myofibroblast differentiation [56].

### Cardiovascular

Peripheral vascular disease, a common and severe complication of diabetes mellitus, is due to a reduction in endothelial progenitor cells. Impaired collateralization of diabetic vasculopathy pathogenesis is not understood. The circulating progenitor cells, and endothelial progenitor cells were decreased 30-40% in diabetes. An inverse correlation was found between endothelial progenitor cells and fasting glucose. Depletion of circulating endothelial progenitor cells may be involved in peripheral vascular complication pathogenesis [57]. Coronary artery disease pathophysiology includes cytokine release and inflammation localized within the vessel wall. Gene expression in circulating blood cells reflects the presence and extent of coronary artery disease in patients undergoing angiography [58]. Circulating endothelial progenitor cells contribute to the regeneration and repair of vessel walls. In older people, maintenance of vascular homeostasis by endothelial progenitor cells may be attenuated due to functional deficits in the cells rather than depletion of CD34/KDR or CD133/KDR cells [59]. Diagnosing obstructive coronary artery disease in at-risk individuals is challenging and often requires both noninvasive imaging methods and coronary angiography. Peripheral blood gene expression can indicate coronary artery disease. A whole-blood test utilizing gene expression and demographic characteristics may be useful for assessing obstructive coronary artery disease in nondiabetic patients [60]. Gene expression alterations in peripheral blood cells can be used to detect the presence and extent of coronary artery disease. RT-PCR analysis of 88 coronary artery disease classifier genes confirmed that diabetic status was the largest clinical factor affecting coronary artery disease associated gene expression changes. Biological pathway and statistical analyses were used to select 113 genes for RT-PCR analysis including coronary artery disease classifiers, cell-type specific markers, and normalization genes. Gene expression correlations identified clusters of coronary artery disease classifier genes which had decreased expression. The final classifier for assessment of obstructive coronary artery disease included 23 genes [28].

### Cancer

**Figure 2:**
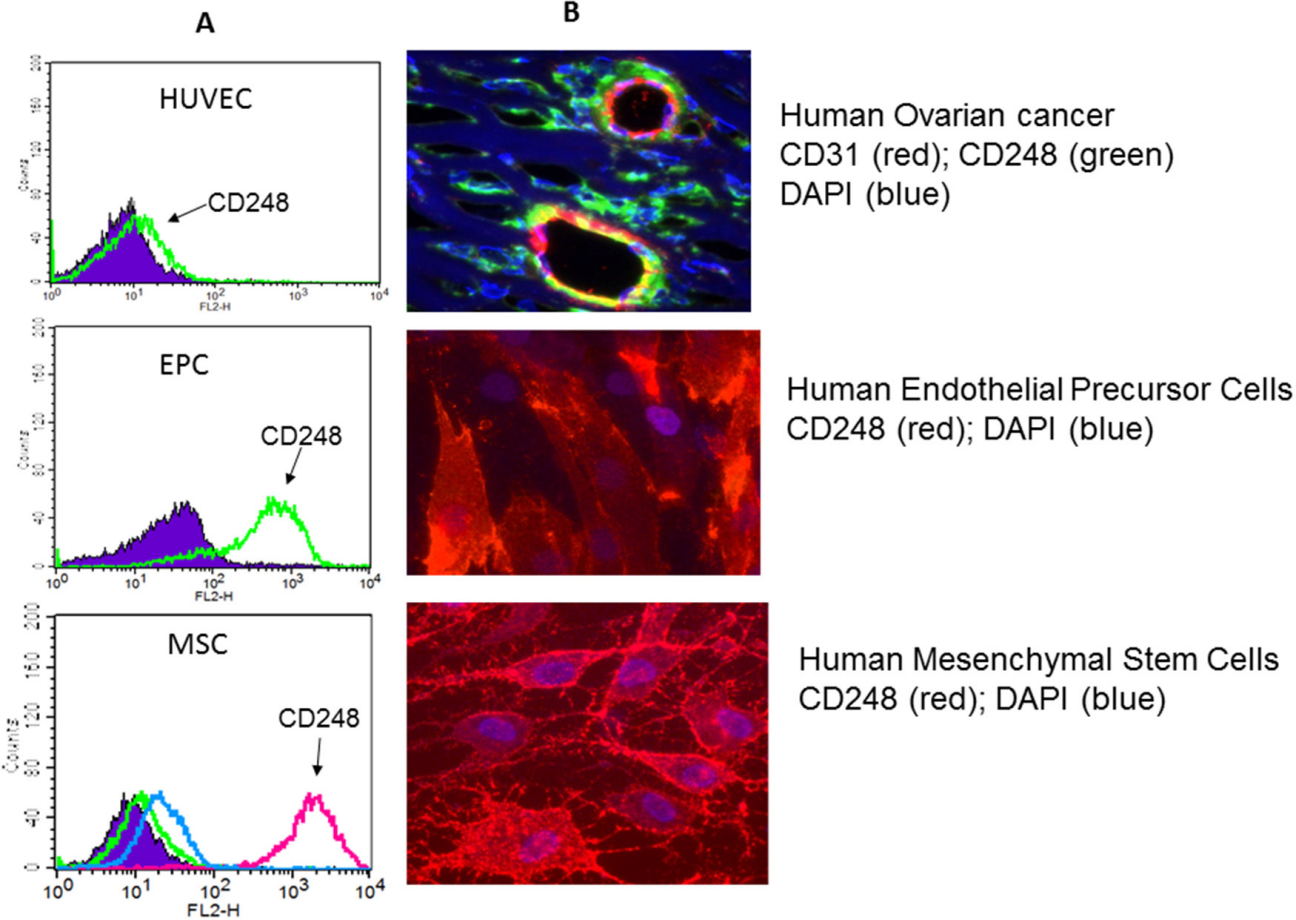
**(A)** Fluorescence activated cell sorting of human umbilical cord endothelial cells (HUVEC), endothelial progenitor cells (EPC) and mesenchymal stem cells (MSC) immunostained for cell surface CD248. **(B)** Panel 1: Immunohistochemical staining of a sample of human ovarian carcinoma with mature blood vessels for CD31 (red), CD248 (green) and DAPI (blue) showing that mature endothelial cells express CD31; pericytes express CD248. Yellow is con-incident staining. Panel 2: Human endothelial cells in culture express CD248 (red); DAPI is blue. Panel 3: Human mesenchymal stem cells in culture express CD248 (red) DAPI is in blue. (adapted from ref. [11]).

In 2000, St. Croix et al. found that the mRNA most upregulated in a sample of human colon cancer vascular cells was the message for CD248 (TEM1) [26, 61, 62]. Later, CD248 was found to be expressed in the vasculature and fibroblasts of human brain tumor specimens, including astrocytoma, anaplastic astrocytoma, glioblastoma multiforme, meningioma, oligodendroglioma, ependymoma and carcinoma brain metastasis [63–65]. By immunohistochemistry, CD248 co-localized with the pericyte marker NG2 in breast cancer specimens but not with the endothelial marker CD31 [21, 22]. In carcinomas, CD248 protein was detected in tumor capillaries and fibroblasts [18]. CD248 stained NG2-positive cells, i.e., pericytes with subcellular localization of CD248 on the surface of the pericyte cell-body and finger-like processes [34, 35, 66–69] (Figure 2). Tumors grow more slowly in CD248/TEM1 knockout mice, suggesting that host CD248/TEM1-positive stroma promotes malignancy [70]. CD248 may play a role in cell-cell adhesion and in adhesion to extracellular matrix proteins [71, 72].

In mice CD248 maps to chromosome 19 and the murine sequence shares 77.5% overall amino acid sequence homology to human CD248 [17]. Using a cDNA probe, murine CD248 mRNA transcripts were detected by Southern blot analysis in many normal tissues particularly those that were collected during embryonic development. In an independent study, Carson-Walter et al. also detected CD248 expression by *in situ* hybridization in the endothelium of developing mouse embryos, notably in the brain and liver [65]. In culture, CD248 expression in murine cells lines analyzed by Northern blot analysis was restricted to embryonic fibroblasts, preadipocytes, and endothelial cells [17]. The function and mechanisms of regulation of CD248 are still incompletely understood. Recently, several new anti-CD248 monoclonal antibodies became available, two recognize the C-type lectin-like domain-Sushi/SCR/CCP and four recognize the sialomucin domain. In addition, a yeast-derived anti-CD248 biobody-78 was developed [4, 69].

**Figure 3:**
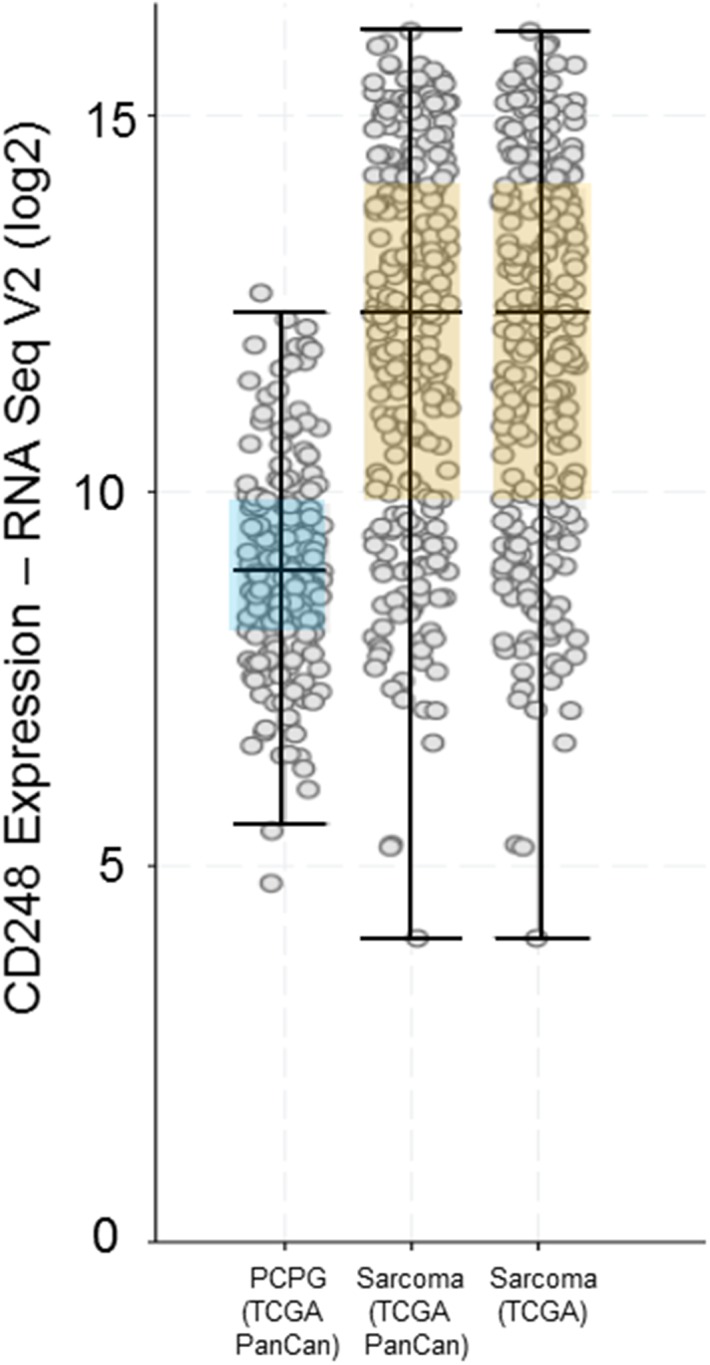
CD248 expression by RNA Seq V2 (log_2_) from the Cancer Genome Atlas (TCGA) showing expression of CD248 in pheochromocytoma and paraganglioma (PCPG) and Sarcoma

The earliest indication that CD248 may be expressed by malignant cells was in a 1992 publication by Rettig et al. who reported immunoreactivity of FB5 antibody in several neuroblastoma cell lines and mentioned FB5+ malignant cells in a subset of sarcoma [15] (Figure 3). Further evidence for CD248 expression by tumor cells came in 2005 with immunostaining of malignant fibrous histiocytoma and liposarcoma showing tumor cell immunoreactivity [18]. Further, CD248 expression was assessed in 86 formalin-fixed, paraffin-embedded human clinical sarcoma specimens. Immunoreactive tissue components were malignant cells, stromal cells and vasculature. Seventy (81%) were positive for CD248, with 44 (51%) reaching at least 50% coverage of immunoreactive tissue components. Staining intensity was scored on the scale 0, 1+, 2+, 3+. All nine sarcoma subtypes tested included specimens with at least 50% immunoreactive tissue components positive with a minimum of 2+ staining intensity, indicating the high prevalence of CD248 in sarcomas [11]. A retrospective analysis of diagnostic reports showed that CD248 was detected in high-grade disease and metastasis. In disseminated human sarcoma xenografts, CD248 protein expression was maintained at different anatomic sites [7–9]. CD248/TEM1 is expressed in stromal cells, endothelial cells and pericytes in various tumors; however, a few studies focused on expression in malignant cells. In 2005, Dolznig et al. showed expression of CD248 transcript in sarcomas, and expression of the protein in malignant cells in one malignant fibrous histiocytoma and one liposarcoma [18]. CD248 protein was found on sarcoma lines, and neuroblastoma lines. A fully human anti-CD248 bound to human A-673 Ewing sarcoma cells and SK-N-AS neuroblastoma cells but not HT-1080 fibrosarcoma cells. Exposure to an anti-CD248 conjugated to saporin was cytotoxic toward only CD248-expressing cells. CD248 expression was assessed in 250 clinical human cancer specimens including 20 cancer subtypes. CD248 expression was mainly perivascular in carcinomas. In sarcoma, CD248 was expressed by malignant cells, perivascular cells, and stromal cells. An anti-CD248 immunotoxin may be a promising therapeutic approach for CD248-positive sarcoma including synovial sarcoma, fibrosarcoma, liposarcoma, and osteosarcoma. A diagnostic/therapeutic targeted therapeutic approach to treatment of CD248-expressing tumors could be promising [10, 11, 73, 74]. In another study, tumor cell CD248 expression was seen in 89% of undifferentiated pleomorphic sarcoma (104/117), 77% adult fibrosarcoma/spindle cell sarcoma (20/26), 62% synovial sarcoma (37/60), 51% leiomyosarcoma (94/185) and 31% rhabdomyosarcoma (39/126) [75]. A cohort soft tissue sarcoma samples were assessed for the correlation between gene expression and protein expression for CD248 and PDGFR-β, a reported interacting protein. CD248 expression was correlated with a better treatment outcome. CD248 expression was highest in liposarcomas and lowest in leiomyosarcomas and was positively correlated with PDGFR-β and heparin sulfate proteoglycan 2 and negatively correlated with carbonic anhydrase IX [76, 77].

**Figure 4:**
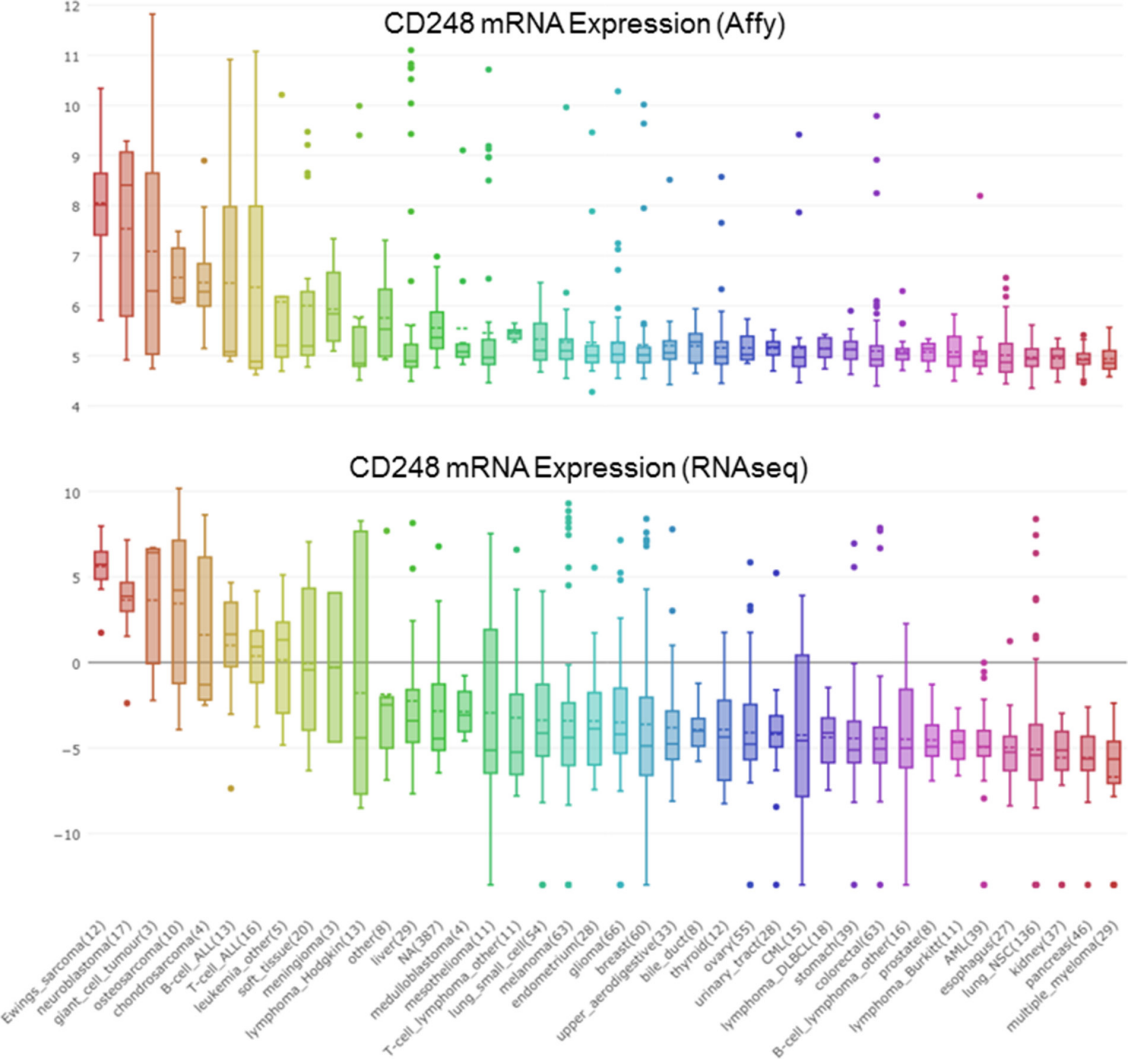
CD248 mRNA expression from cBioPortal in a wide variety of tumors as determined by Affymatrix array and by RNASeq

Amongst central nervous system tumors, glioblastoma multiforme are the most aggressive subclass. Glioblastoma multiforme have massive neovascularization. Although CD248 is not expressed in normal human adult brain, it is expressed in the angiogenic vasculature of high-grade glioma. CD248 is not expressed by the glioma endothelial cells. It is expressed by the perivascular cells [3, 78] (Figure 4). Gliomas are characterized by profound local immunosuppression. Glioma-derived pericytes are characterized by the expression of CD90, CD248, and platelet-derived growth factor receptor-β. Glioma-derived pericytes expressed prostaglandin E synthase, inducible nitric oxide synthase, human leukocyte antigen-G, hepatocyte growth factor and transforming growth factor-β (TGF-β). Thus, human cerebral CD90+ perivascular cells have T cell inhibitory capability, a critical role in tumor vascularization, and may promote local immunosuppression in malignant gliomas [79]. Glioblastoma multiforme is characterized by malignant cell heterogeneity and a complex tumor microenvironment. Mixed cell cultures from gliomas expressing high CD56, SOX2, SOX9, and low CD105, CD248, αSMA are tumorigenic and express cancer stem cell markers [80].

CD248 is expressed in fibroblasts and pericytes of colorectal cancer (Figure 4). To determine whether CD248 expression was increased by radiotherapy, CD248 expression in rectal cancers, normal mucosa, including distant and adjacent normal mucosa, and primary tumors was assessed. CD248 expression in stroma increased from normal mucosa to tumor both in radiotherapy-treated and non-radiotherapy-treated tumors. However, no direct relationship between CD248 expression and response radiotherapy was found [81]. DNA aberrations involved in colorectal cancer correlate with MSI status. MSI-H tumors tend to have a low number of chromosomal aberrations and high DNA methylation frequency. Highly expressed markers include CD248, ERCC6, ERGIC3, GNAS, MMP2, NF1, P2RX7, SFRS6, SLC29A1 and TBX22 [82]. Astrocyte-elevated gene-1 (AEG-1) is expressed in various human cancers including cervical carcinoma. AEG-1 knockdown by shRNA inhibited SiHa cervical carcinoma cell proliferation and decreased expression of angiogenesis-related genes HIF-1α, Tie2, VEGF and CD248 by the cells [83].

Conventional chemotherapy fails in ovarian cancer due to development of drug resistance. To focus cytotoxic drug impact to tumor cells, polymeric nanoparticles with a CD248-targeting antibody were engineered with solubilizing agent polyethylene glycol. The biological impact was evaluated in tumor-associated endothelial cells, primary lymphocytes, and epithelial ovarian cancer OVCAR-5 cells. Fluorescence microscopy and flow cytometry analysis confirmed interaction of the nanoparticles with CD248-positive cells, but not with CD248-negative cells [84].

Gastric cancer is a major cause of cancer mortality worldwide. Using whole-genome DNA microarrays, mRNA from human gastric cancer samples was compared to adjacent healthy mucosa. Genes highly expressed in gastric cancer were CD248, NSDl, RABl7, ABCG8, Ephbl and P2RY2. P2RY2, Ephbl and CD248 were explored for gastric cancer detection [85]. The cross talk between tumor cells and the surrounding stroma contributes to hepatocellular carcinoma progression. Activation of hepatic stellate cells during liver damage induces CD248 expression, which contributes to regulating the balance of liver regeneration and fibrosis (Figure 4). Histological analysis of human hepatocellular carcinoma samples indicated an inverse correlation between tumor cell proliferation and stromal CD248 expression. Genetic inactivation of CD248 resulted in accelerated tumor growth in an inducible mouse hepatocellular carcinoma model. Thus, CD248-expressing hepatic stellate cells are a negative regulator of hepatocellular carcinoma progression [86].

Metastasis is a multistep process that is critically dependent on the interaction of metastasizing tumor cells with cells in the local microenvironment. Within tumor stroma, pericytes and myofibroblasts have upregulated CD248 expression. Comparative experiments in wild-type and CD248-deficient mice showed that stromal CD248 promoted spontaneous metastasis. CD248-expressing pericytes in the tumor facilitated distant site metastasis by promoting tumor cell intravasation in a cell contact–dependent manner, resulting in higher circulating tumor cells. In independent cohorts of primary human breast cancers, upregulated CD248 expression correlated with increased metastasis [87].

### CD248 imaging

The Hoechst 33342 exclusion side population assay is a method used to identify cells with stem cell-like properties in both carcinomas and sarcomas. The molecular profile of sarcoma side population cells is poorly understood. Five sarcoma lines (HT-1080 fibrosarcoma, SJSA-1 and HOS osteosarcoma, A-673 and SK-ES-1 Ewing sarcoma) were used for the side population analysis. CD248 was expressed in sarcoma side population cells, thus, supporting the hypothesis that CD248 is a therapeutic target for sarcoma [9]. In another study, primary human osteosarcoma samples had 3.9% side population cells which overexpressed ABC transporters, including ABCA1, ABCB1, ABCB2 and ABCG2 that contribute to multi-drug resistance. The osteosarcoma side population cells had high CD248 and CD133, OCT3/4A, Nanog and Nestin, which are responsible for high self-renewal and deregulated cell proliferation. Upon proliferation, CD248 high osteosarcoma side population cells regenerated the tumor population [88, 89]. Some sarcoma subtypes, such as Ewing sarcoma, have characteristics of neuroendocrine differentiation. Cancers with neuroendocrine properties and/or neuroectodermal origin, including neuroblastoma, small cell lung cancer and melanoma, were assessed for CD248 in human clinical specimens and in human cell lines. In human clinical specimens, vascular CD248 staining was observed in neuroblastoma, small cell lung cancer and melanoma. Malignant cell staining was strongest in neuroblastoma, weak in melanoma and rare in small cell lung cancer. In cell lines, CD248 was detected in neuroblastoma lines, including cancer stem cell-like side population cells, but was absent in melanoma and was rare and weak in small cell lung cancer. Human neuroblastoma xenograft tumors were positive for CD248 suggesting that CD248 may be a therapeutic target for neuroblastoma [7].

CD248 is expressed on tumor-associated microvascular pericytes, tumor-associated stromal cells and on malignant cells of mesenchymal origin. A humanized anti-CD248 monoclonal antibody called ontuxizumab (MORAb-004) was tested in preclinical and clinical studies. MORAb-004 (Ontuxizumab), an anti-CD248 humanized monoclonal antibody, is in sarcoma clinical trials. Development of positron emission tomography for CD248 expression may allow stratification of patients, potentially enhancing outcomes for patients with CD248 expressing tumors. Biodistribution studies in xenograft-bearing mice confirmed high tumor uptake ^89^Zr-Ontuxizumab can be used to determine CD248 expression. Reliable CD248 PET imaging in sarcoma patients may allow identification of patients that can gain the greatest benefit from anti-CD248 therapy [90–92].

An extensive panel of recombinant CD248 protein extracellular domain fragments and novel antibodies against CD248 extracellular protein motifs were developed [16, 93]. An analysis of CD248 expression in metastatic melanoma specimens was conducted to determine the potential of CD248 as a therapeutic target. CD248 expression assessment was performed on a tumor microarray with 136 Stage IV and 33 paired Stage III melanoma specimens. BRAF mutation was evaluated in the melanoma specimens as well. CD248 was present in 70% of the melanoma specimens. CD248 and BRAF mutation expression was similar in stage III and IV specimens. CD248 expression was present in 86% of the stage IV tumor microarray specimens, and there was no expression in the normal tissue controls [94]. The expression of CD248 and pathway-associated proteins was examined for association with 5-year disease-specific survival of a colorectal cancer cohort divided into training and validation sets. Stromal CD248 expression was prognostic; however, a stronger prognostic signature included expression scores for CD248 and HIF2α in stroma and vessels. The expression of these markers was associated with decreased survival in the training set and the validation set. Prognostic score derived predicted survival in a colorectal cancer Stage II patient cohort [93].

### CD248 clinical trials

CD248 is a transmembrane protein on activated mesenchymal cells including tumors. A humanized monoclonal antibody, MORAb-004 targeting CD248, was the first agent to enter clinical development for this target. The first-in-human, open-label, Phase I study recruited patients with treatment-refractory solid tumors. MORAb-004 was administered intravenously once weekly in 4-week cycles. The goals were determination of safety for multiple MORAb-004 infusions, determination of maximum tolerated dose, pharmacokinetics, detection of any anti-human antibody response, and assessment of objective radiographic response to therapy. Thirty-six patients were treated at 10 MORAb-004 dose levels from 0.0625 to 16 mg/kg. Drug-related adverse events were mainly grade 1–2 infusion toxicities. Dose limiting grade 3 vomiting occurred at 16 mg/kg. Eighteen of 32 evaluable patients across all doses achieved disease stability, with minor radiographic responses in 4 patients (pancreatic neuroendocrine, hepatocellular, and sarcoma tumor types). MORAb-004 accumulation began at 4 mg/kg and saturable elimination began at 0.25 mg/kg. Exposure increased in a dose proportional manner with terminal half-life increasing proportionally with dose. The maximum tolerated dose was 12 mg/kg. Preliminary antitumor activity was observed. Safety profile, pharmacokinetics, and early antitumor activity suggested that MORAb-004 should be studied further for efficacy (NCT01773434) [94, 95]. A randomized, double-blind, placebo-controlled, phase II study evaluated the safety and efficacy of anti-CD248 ontuxizumab (MORAb-004) in patients with chemo-refractory metastatic colorectal cancer. Patients (126) received weekly intravenous ontuxizumab (8 mg/kg) or placebo plus best supportive care until progression or toxicity. There was no difference in progression-free survival, overall survival or overall response rate between the ontuxizumab and placebo groups. The treatment-related adverse events were fatigue, nausea, decreased appetite and constipation. Ontuxizumab was well tolerated (NCT01507545) [96]. A Phase II study evaluated progression-free survival, pharmacokinetics, and tolerability of 2 doses of ontuxizumab in metastatic melanoma patients. Metastatic melanoma patients having received at least 1 prior systemic treatment were randomized to receive ontuxizumab (2 or 4 mg/kg) weekly until disease progression. The 24-week progression free survival, was the same for all patients (8.3 weeks). One patient receiving 4 mg/kg had a partial response. Of the response evaluable patients, 27/66 or 40.9% had stable disease. The median overall survival was 31.0 weeks. Efficacy of single-agent ontuxizumab at these doses in melanoma was low (NCT01335009) [97]. A Phase I trial of ontuxizumab (MORAb-004) was conducted in children with relapsed or refractory solid tumors by the Children’s Oncology Group Phase I Pilot Consortium. Ontuxizumab was administered intravenously on days 1, 8, 15, and 22 of a 28-day cycle at three dose levels (4, 8, and 12 mg/kg). Following determination of the maximum tolerated dose and recommended Phase II dose, an additional cohort of six patients (<12 years) was enrolled for pharmacokinetics evaluation. Twenty-two patients including patients with neuroblastoma, Ewing sarcoma, rhabdomyosarcoma, and other tumors, were fully evaluable. Dose dependent clearance was like the adult value at 12mg/kg. Ontuxizumab administered weekly at 12 mg/kg appears to be well tolerated in children with relapsed or refractory solid tumors (ADVL1213) [98].

Based upon a Phase I study conducted in sarcoma patients, ontuxizumab received FDA orphan drug designation for sarcoma (NCT00847054). The MORAb-004-203-STS Phase II Study, a randomized, double-blind, placebo-controlled study, examined the safety and efficacy of ontuxizumab in combination with gemcitabine and docetaxel in four subtypes of metastatic soft tissue sarcoma. The primary endpoint was progression-free survival. The overall survival benefit, biomarker identification, and safety of the treatment regimen were assessed (NCT01574716).

### CD248-targeting experimental therapeutics

CD248 has value as a tumor vascular marker. Anti-CD248 imaging can detect and monitor tumor responses and select patients who may benefit from CD248-targeted therapies. Antibody drug conjugates provide a way to increase the therapeutic value of an antibody while decreasing the dose of the antibody. The requirements for a cell surface molecule to be suitable as an antibody-drug conjugate (ADC) target are well-established [99–106]. The optimal ADC has antigen recognition that is not different from the unconjugated antibody. ADCs usually include 2-4 highly potent anticancer agent small molecule drugs. The covalent linker that tethers the antibody to the small molecule drug must be stable in plasma and labile when internalized by the target cell.

The drugs often used in ADCs, maytansines and dolastatins, target microtubules. The dynamic flux of microtubules is a key target of anticancer therapies. Although principally recognized in mitotic function for their role in separating the duplicate set of chromosomes during cell division, microtubules are an essential cytoskeleton component and are critical in directional transport of proteins and organelles, maintenance of cell motility, cell shape and scaffolding, intracellular transport, secretion, neurotransmission and relay of signaling between cell surface receptors and the nucleus [107, 108]. The biologic function of microtubules relies on the assembly and disassembly dynamics of tubulin polymerization [109, 110].

An anti-endosialin-MC-VC-PABC-monomethyl auristatin E (MMAE) ADC was prepared and assessed in cell culture and in two human tumor xenograft models, demonstrating high specificity and profound, durable antitumor efficacy. The antibody–drug conjugate anti-CD248-MC-VC-PABC-MMAE, with 3–4 MMAE molecules per ADC, was selectively cytotoxic to CD248-positive cells in culture and produced profound, durable tumor control in human CD248-positive tumor xenografts. The cytotoxicity of anti-CD248-MC-VC-PABC-MMAE was assessed in human cell lines with varied CD248 levels. SK-N-AS neuroblastoma and the A-673 Ewing sarcoma xenografts were selected for *in vivo* efficacy testing. The treatment groups included a vehicle control, unconjugated anti-CD248, an admix control consisting of anti-CD248 and free MMAE, and the anti-CD248-MC-VC-PABC-MMAE conjugate. The unconjugated anti-CD248 had no antitumor activity. The anti-CD248-MC-VC-PABC-MMAE conjugate produced marked prolonged tumor responses in both models. These proof-of-concept results break new ground and open an approach to these rare, neglected tumors [111]. In another study, the preclinical efficacy of a novel antibody-drug conjugate, consisting of a humanized CD248 monoclonal antibody, hMP-E-8.3, conjugated to a potent duocarmycin derivative was tested in CD248 expressing cancer cell lines, and had specific, target-dependent killing activity. High CD248 expression in cells correlated with efficient internalization and cytotoxic effects *in vitro*. In an osteosarcoma xenograft study, treatment led to a long-lasting tumor growth inhibition [112].

Near infrared optical imaging with specific antibodies can provide information without use of radioactivity. A panel of human, multivalent Fc-fusion proteins based on a single chain antibody (scFv78) that recognizes both human and mouse CD248 was developed. The biodistribution of the selected Fc-fusion protein had minimal binding to normal organs. The near infrared imaging and tomography results suggested that the selected Fc-fusion-NIR tracer performed well in distinguishing CD248 expressing tumor grafts from normal organs and control grafts *in vivo* [113].CD248 expression was analyzed in clinical sarcoma specimens processed by standard formalin-fixed paraffin embedded techniques collected including 19 human sarcoma subtypes and 8 human sarcoma cell lines. Near-infrared imaging of tumor-bearing mice was used to validate the selected Fc-fusion protein binding to CD248 expressing sarcoma *in vivo*. CD248 expression was identified in 96% of human sarcomas, of which 81% expressed CD248 both on tumor cells and the tumor vasculature [114]. In addition to application in molecular imaging, the selected Fc-fusion protein can be used in immunotoxin-based therapy and nanoparticle therapy. The selected Fc-fusion protein and selected Fc-fusion protein-labeled nanoparticles rapidly internalized after specific CD248 binding. The selected Fc-fusion protein-saporin immunoconjugate produced concentration-dependent cytotoxicity in CD248-positive cells in culture. Specific CD248-positive tumor localization of the selected Fc-fusion protein was confirmed with optical imaging [115].

To determine whether CD248 has potential as a vaccine antigen, immunocompetent mice were immunized with CD248 cDNA fused to the minimal domain of the C fragment of tetanus toxoid (TT) to form a vaccine. CD248-TT vaccination elicited CD8+ and/or CD4+ T cell responses against immunodominant CD248 protein sequences. Prophylactic immunization of mice with CD248-TT prevented or delayed tumor formation in mice. Vaccination of tumor-bearing mice decreased tumor vascularity, increased CD3+ T cell infiltration, and slowed tumor progression. CD248-TT vaccination elicited CD8+ cytotoxic T cell responses against tumor-specific antigens indicating that targeting CD248 has therapeutic potential in cancer immunotherapy [116]. A DNA-based vaccine approach demonstrated that CD248 can be effectively targeted immunologically; anti-tumor responses were generated in several mouse models; and CD8+/CD4+ T cell responses were elicited against peptides derived from CD248 protein. Thus, CD248 is a novel immunotherapeutic target for cancer treatment and using a translatable DNA-based immunotherapy [117]. Mice lacking CD248 were immunized with (4-hydroxy-3-nitrophenyl)acetyl chicken γ-globulin to examine the role of CD248 in popliteal lymph node expansion and subsequent immune responses. CD248 was required for complete popliteal lymph node expansion but not for co-ordination of B and T cell compartmentalization or antibody production following (4-hydroxy-3-nitrophenyl)acetyl chicken γ-globulin immunization. CD248 expression in human MG63 osteosarcoma cells and mouse embryonic fibroblasts produced a pro-proliferative and pro-migratory phenotype. CD248 is involved in secondary lymphoid organ remodeling during adaptive immune responses [118]. CD248 is expressed by human, but not mouse (C57BL/6), CD8+ naive T cells and is found only on CD8+ CCR7+ CD11a low naive T cells and on CD8 single-positive T cells in the thymus. CD248 transfection into MOLT-4 T-cells with CD248 cDNA decreased cell proliferation. CD248 knock-down on naive CD8 T cells increased cell proliferation. Thus, CD248 has opposing functions on hematopoietic (CD8+) versus stromal cells and suggests that CD248 has a role in maintaining naive CD8+ human T cells in a quiescent state [5]. T cell functional capacity is most affected by age and is linked to decreased response to infections and impaired differentiation. Comparison of age-related DNA methylation changes and gene expression in CD4+ and CD8+ T cells from younger and older individuals indicated a difference between T cell subsets, with increased methylation changes and higher methylome variation in CD8+ T cells with age, an age-related epigenetic change. There was an inverse correlation between methylation and expression of genes associated with T cell mediated immune response among these was CD248 [118]. Thymic cortical and medullar microenvironments are essential for thymocytes maturation. Mesenchymal stem cells engraft into thymic tissue and continue to express CD248 and other proteins essential for thymic development. By this mechanism mesenchymal stem cells provide a microenvironment for the reconstitution and functional maturation of the thymus and increase the understanding of mesenchymal stem cells therapeutic efficacy in several autoimmune diseases [119].

CD248 has been described as a tumor antigen suitable for chimeric antigen receptor (CAR) T cell therapy. The therapy involves depleting B cells in combination with the use of a cell comprising a chimeric antigen receptor (CAR) that targets a tumor antigen and includes using a cell comprising a CAR that targets a B cell antigen [120]. A subset-optimized CART cells and related methods. CD4 + and CDS+ T cells that express CARs containing specific combinations of intracellular signaling domains can be used to increase persistence and anti-tumor activity of the infused CAR-expressing T cells [121].

## CONCLUSION

Cd248, first identified in 1992 as the antigen for the FB5 antibody, has continued to interest investigators interested in the elucidation of disease mechanism(s) and as a therapeutic target in a wide variety of fibrotic conditions and cancer. The function of CD248 has not been fully elucidated; however, the tissue distribution and clear pattern of upregulation in pathology, and expression on the cell surface has made CD248 a potential biomarker, target of imaging agents and target for therapeutics. Although no investigational agents targeting CD248 have yet reached FDA approval, clinical trials are underway.
